# Cloning and Polymorphisms of Yak Lactate Dehydrogenase b Gene

**DOI:** 10.3390/ijms140611994

**Published:** 2013-06-05

**Authors:** Guosheng Wang, Xingbo Zhao, Juming Zhong, Meng Cao, Qinghua He, Zhengxin Liu, Yaqiu Lin, Yaou Xu, Yucai Zheng

**Affiliations:** 1 College of Life Science and Technology, Southwest University for Nationalities, Chengdu 610041, China; E-Mails: phoenix.1984@163.com (G.W.); demeatry@gmail.com (Q.H.); liuruii@sohu.com (Z.L.); linyaqiu0001@yahoo.com.cn (Y.L.); xuyaou@163.com (Y.X.); 2 College of Animal Science, China Agricultural University, Beijing 100193, China; E-Mails: zhxb@cau.edu.cn (X.Z.); caomengbi@163.com (M.C.); 3 College of Veterinary Medicine, Auburn University, Auburn, AL 36849, USA; E-Mail: zhongju@auburn.edu

**Keywords:** *Bos grunniens*, lactate dehydrogenase, polymorphism, hypoxic adaptation

## Abstract

The main objective of this work was to study the unique polymorphisms of the lactate dehydrogenase-1 (LDH1) gene in yak (*Bos grunniens*). Native polyacrylamide gel electrophoresis revealed three phenotypes of LDH1 (a tetramer of H subunit) in yak heart and *longissimus* muscle extracts. The corresponding gene, *ldhb*, encoding H subunits of three LDH1 phenotypes was obtained by RT-PCR. A total of six nucleotide differences were detected in yak *ldhb* compared with that of cattle, of which five mutations cause amino acid substitutions. Sequence analysis shows that the G896A and C689A, mutations of *ldhb* gene, result in alterations of differently charged amino acids, and create the three phenotypes (F, M, and S) of yak LDH1. Molecular modeling of the H subunit of LDH indicates that the substituted amino acids are not located within NAD^+^ or substrate binding sites. PCR-RFLP examination of G896A mutation demonstrated that most LDH1-F samples are actually heterozygote at this site. These results help to elucidate the molecular basis and genetic characteristic of the three unique LDH1 phenotypes in yak.

## 1. Introduction

Yak (*Bos grunniens*) inhabits the Qinghai-Tibet Plateau and is a unique cattle species adapted to the cold, hypoxic environment (altitude of 2000–5000 m). It is also the most important domestic animal native to the Qinghai-Tibet Plateau and provides the local farmers with products such as meat, milk, and hair. The total population is thought to be around 14 million, of which 13 million are distributed in China [1]. The physiological and molecular mechanisms of the yak’s adaptation to the hypoxic habitat are of great interest in yak research.

Lactate dehydrogenase (LDH, EC 1.1.1.27) is the key enzyme in anaerobic glycolysis, catalyzing the conversion between pyruvate and lactate, and playing an important role in energy metabolism [2]. LDH is a tetramer composed of M and H subunits encoded by *ldha* and *ldhb* genes respectively [3]. Assembly of H and M subunits results in five electrophoretically distinguishable LDH isozymes (LDH1 to LDH5, also as H4, H3M, H2M2, HM3, and M4, respectively) in somatic tissues of nearly all vertebrates. LDH is an important parameter in the study of anaerobic metabolism. A higher total LDH activity in heart and skeletal muscles has been reported in pikas from high altitudes when compared to pikas from low altitudes, which helps the pikas in high altitudes to improve anaerobic activity and to enhance lactate removal in muscles [4], since high LDH activity can catalyze more pyruvate to produce lactate in skeletal muscle, while in heart tissue it can catalyze the reverse reaction under aerobic condition to remove lactate.

Yaks exhibit polymorphism in the *ldhb* gene as a result of several nucleotide mutations, leading to the formation of different H subunits, and thus different LDH1 (composed only of H subunits). The *ldhb* gene, or LDH1 polymorphism, has been revealed in several yak breeds by isozyme electrophoresis or gene cloning [5,6], but has not been observed in other cattle species. Whether there is a relationship between yak *ldhb* polymorphism and hypoxia adaptation is unclear. It is now recognized that acclimation to severe hypoxia decreases the mitochondrial content of muscle fibers, and shifts the oxidative muscle metabolism towards a higher reliance on carbohydrates as fuel [7]. LDH is required for the utilization of carbohydrate when oxygen supply is not enough, and it was reported that yaks contained higher LDH activity in *longissimus* muscle than cattle [8]. We previously purified three genetic variants of LDH1 from heart tissues of Jiulong yaks, and found differences in the *Km* values of LDH1 variants [9]. Based on the specific hypoxic habitat of yaks, and the key role of LDH in anaerobic metabolism, we hypothesized that the occurrence of *ldhb* genetic polymorphisms in yak is probably related to the hypoxic condition during hypoxia acclimation, and thus cloned *ldhb* gene from heart tissues of Jiulong yaks to provide molecular evidences for the hypoxic adaptation of yak.

## 2. Results

### 2.1. Phenotypes of Yak LDH1 Isozyme

**Figure 1:**
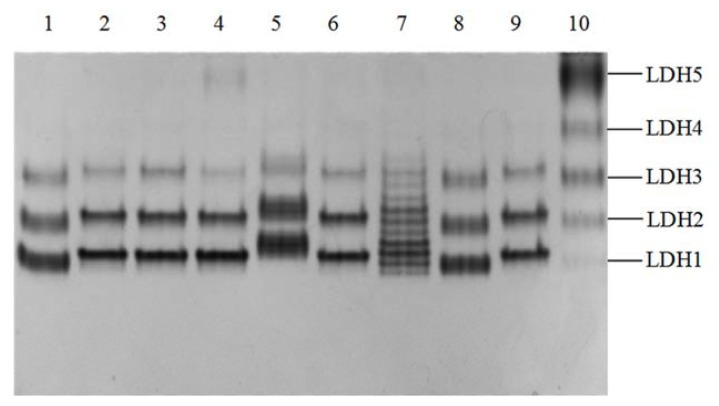
Native PAGE of LDH isozymes in yak heart and skeletal muscles. LDH1-F: lane 1, 8, 10; LDH1-M: lane 2, 3, 4, 6, 9; LDH1-S: lane 5; multiple band phenotype of LDH isozymes: lane 7. Lane 1 to 9: heart tissues, lane 10: *longissimus* muscle.

LDH isozymes in heart and *longissimus* muscle extracts of Jiulong yaks exhibited five bands (LDH1 to LDH5) on native polyacrylamide gel electrophoresis (PAGE). LDH1 (tetramer of four H subunits) showed polymorphisms. Three phenotypes of LDH1 were observed and named LDH1-F, LDH1-M, and LDH1-S respectively according to their different gel mobilities (Figure 1). Similar differences also existed in LDH2 (H3M) to LDH4 (HM3) isozymes. Most LDH1-F or LDH1-S phenotypes exhibited obvious broad bands compared with those of LDH1-M phenotype (Figure 1, lane 1, 5, and 8). These broad bands were in fact composed of several fine bands of corresponding LDH isozymes. In addition, a multiple band phenotype of LDH isozymes was observed in a yak as shown on native PAGE with LDH activity staining (Figure 1, lane 7). In 130 yak samples assayed, 62 were LDH1-F, 63 were LDH1-M, 4 were LDH1-S, and 1 was a multiple band phenotype.

### 2.2. Cloning and Sequencing of Yak *ldhb* cDNA

To reveal the molecular basis of *ldhb* polymorphism, *ldhb* gene associated to yak LDH1-F, LDH1-M, and LDH1-S phenotypes was cloned by RT-PCR, and the three obtained cDNA sequences were submitted to GenBank with accession number of HQ874652, HQ874653, and HQ874654, respectively. The three *ldhb* alleles encode the H subunit of LDH, with a length of 333 amino acids (excluding the methionine at the N terminal).

By cloning and sequencing the *ldhb* gene of several yaks, a total of six nucleotide differences were detected in yak (Table 1), and five of which cause amino acid substitutions (Table 2). Changes of G to A at the 896 nucleotide position and C to A at the 689 nucleotide position result in substitutions of differently charged amino acids (Arg to Gln at the 298 amino acid position, and Glu to Ala at the 229 amino acid position, respectively), which contribute to the formation of LDH1-F and LDH1-S phenotypes due to the significant changes in their isoelectric point (pI) values (Table 3). *ldhb*-F, *ldhb*-M, and *ldhb*-S sequences of yak are similar to that of bovine *ldhb* (GenBank accession no. BC102217.1), except the existence of a nucleotide difference or several single nucleotide polymorphisms (Table 1). There existed little difference in molecular weight of H subunits from the three LDH1 phenotypes (Table 3).

When *ldhb* cDNA was cloned using the specific sample showing multiple LDH bands in Figure 1 (lane 7), both *ldhb*-F and *ldhb*-S sequences were obtained, indicating that two types of H subunits (defined as Hf and Hs, respectively) contributed to the formation of LDH isozymes in the sample. LDH1 (H4) in this sample actually consists of five forms (Hf4, Hf3Hs, Hf2Hm2, HfHm3, and Hm4), LDH2 (H3M) consists of four forms (Hf3M, Hf2HsM, HfHm2M, and Hm3M), and LDH3 (H2M2) consists of three forms (Hf2M2, HfHsM2, and Hm2M2). These bands could be well separated on the gel.

### 2.3. Genotyping of *ldhb* G896A in Two Yak Breeds

**Figure 2:**
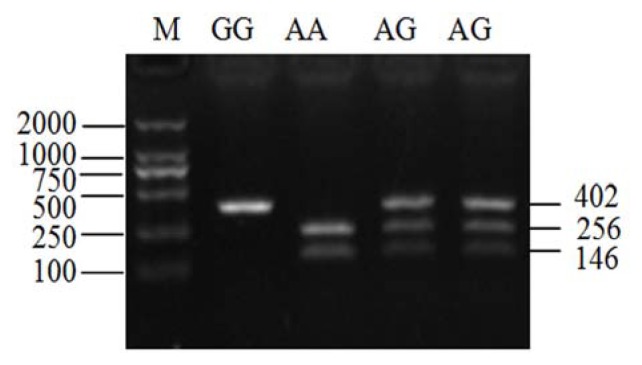
PCR-RFLP analysis of *ldhb* G896A mutation. M: DL2000 DNA marker (bp). PCR products were digested by *Taq I* and separated by agarose electrophoresis. Genotypes are indicated on the top of each lane.

*ldhb* G896A substitution in yak population was assayed by PCR-RFLP, and three genotypes were detected (Figure 2). According to their influence on electrophoresis profile, the genotype *ldhb*-AA and *ldhb*-AG are associated with LDH1-F phenotype, and *ldhb*-GG with LDH1-M or LDH1-S phenotypes. In both yak breeds studied, yaks carrying the *ldhb*-AG genotype accounted for around 40% to 50% of the total (Table 4), indicating that most of the samples exhibiting LDH1-F phenotype are, in fact, composed of heterologous H subunits encoded by *ldhb*-A and *ldhb*-G alleles. However, the present genotyping method cannot distinguish between the genotypes of the LDH1-M and LDH1-S phenotypes, since their *ldhb* genes have the same nucleotide at position 896. The LDH1-S phenotype was identified by electrophoresis of LDH isozymes, as shown in Figure 1, or direct sequencing fragment containing nucleotide position 689.

### 2.4. Molecular Modeling of H Subunit of Yak LDH

**Figure 3:**
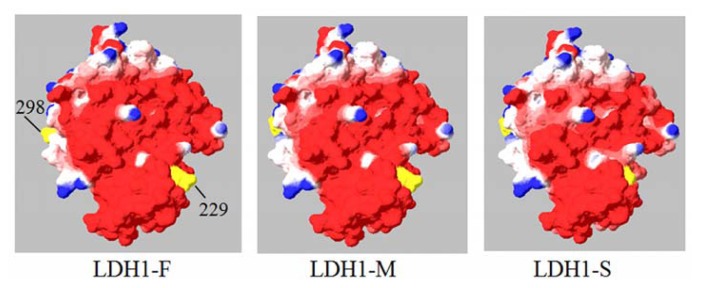
Charge distribution of H subunit of yak LDH1 variants. Red color represents negative charge, blue represents positive charge; the two mutations are labeled with a bright yellow color (298 Gln/Arg and 229 Glu/Ala). The areas of red color decreased from LDH1-F, LDH1-M, to LDH1-S.

Molecular modeling analysis showed that His193 and Thr248, as well as Arg106, are substrate binding and catalytic sites of LDH, which are conserved in all three LDH1 phenotypes of yak. Among the five amino acid substitutions, Arg/Lys18 and Glu/Ala230 are partially exposed, Gly/Val136 and Val/Met234 are entirely buried, and a small portion of Arg/Gln299 is exposed. All these sites are not located within NAD^+^ or substrate binding sites. The negative charges are decreased in the order of LDH1-F, LDH1-M, and LDH1-S phenotypes (Figure 3) as a result of amino acid substitution.

## 3. Discussion

To date, no bovine species other than yaks has been reported to display the occurrence of *ldhb* genetic polymorphisms. We have previously identified two phenotypes of LDH1 in Maiwa yaks (a yak breed in China) by gene cloning [6], and purified three phenotypes of LDH1 in Jiulong yaks [9]. In this study we obtained three *ldhb* cDNA sequences, encoding H subunits, of the three LDH1 phenotypes of Jiulong yaks. The mutations of *ldhb* G896A and C689A result in amino acid changes (Gln298Arg and Glu229Ala) and significant decrease in negative charges of LDH1-F, LDH1-M, and LDH1-S phenotypes of Jiulong yaks, which can well explain their mobility differences on PAGE profiles.

Based on the tetramer combination of LDH isozymes, we previously proposed that the occurrence of genetic polymorphisms of H subunit of LDH might give rise to 5 to 2 isozyme forms for LDH1 to LDH4, respectively [6]. In this study, we observed clearly the existence of multiple bands of LDH1 to LDH3 isozymes (Figure 1, lane 7). cDNA cloning of this sample demonstrated that its H subunit is encoded by two *ldhb* alleles (*ldhb*-F and *ldhb*-S). Meanwhile, cDNA cloning and PCR-RFLP analysis also revealed both *ldhb*-F and *ldhb*-M alleles, which encode two different H subunits and cause the broad bands of LDH1 and LDH2 on the gel (Figure 1, lanes 1 and 8) when they form the LDH isozymes in the tetramer combination. The electrophoresis and activity staining of yak LDH isozymes were not sufficient to clearly distinguish the isozyme formed by two H subunits, encoded by *ldhb*-F and *ldhb*-M or *ldhb*-S and *ldhb*-M, exhibiting only one broad band for LDH1 (also LDH2, and LDH3) on the gel as shown in Figure 1. However, the PCR-RFLP protocol can accurately determine the genotypes of *ldhb* at position 896, which specify LDH1-F and LDH1-M/LDH-S phenotypes (Table 4). The reason for the clear separation of multiple LDH bands in lane 7 of Figure 1 is mostly due to the great difference in charge/mass between the Hf and Hs subunits, while the combination of Hf and Hm, or Hm and Hs subunits will result in broad LDH1 bands. Our results demonstrated that most LDH1-F samples are herterologous at the *ldhb* 896 position (58 in 63 samples) and are actually composed of both LDH1-F and LDH1-M bands (Table 4). The detection of mutation at nucleotide position 689 of *ldhb* needs to be developed, although the occurrence is much lower than that of nucleotide position 896.

The yak has high similarities with domestic cattle (*Bos taurus*) in amino acid sequence of proteins assayed [10,11]. Thus, yak would be a suitable model to study the molecular adaptation to hypoxia in bovine species. Hypoxic adaptation is very complicated and is related to many molecules [12–15]. Compared to low-altitude inhabitants, the skeletal tissue adaptation to hypoxia in Tibetans is attributed to the decrease of oxidative capacity and the low capillary density of muscle [16]. In addition, it has been reported that the expression of skeletal muscle LDH and glycerol 3-phosphate dehydrogenase is relatively low in Tibetans adapted to high altitude [17]. This seems to be different from other reports [4], and different mechanisms might be involved. However, most of studies show that the adaptation in metabolism is attributable to the decrease of muscle oxidative capacity, although there are variations in this trend. Yaks may have a similar mechanism for hypoxic adaptation. The identification of genes subject to positive selection is very important [12]. However, whether the mutations in the yak *ldhb* gene have a relationship with the hypoxic environment needs further investigation.

The functions of different LDH1 phenotypes of yak are unclear. We reported that yak LDH1-M phenotype exhibited a similar *Km* (NADH), and the same mobility on a gel as bovine LDH1, and the LDH1-F phenotype showed a significantly higher *K*m value for NADH and a lower *K*m value for pyruvate than yak LDH1-M and LDH1-S, as well as bovine LDH1 [9]. This suggests that LDH1-F is more suitable for catalyzing the conversion of pyruvate to lactate, and this will be of significance for energy production under hypoxic conditions. Nearly 50% of Jiulong yaks carry *ldhb*-AA or *ldh*b-AG genotypes, which are related to LDH-F1, and thus may enhance their adaptation to hypoxia. Results from the present study proved that yak *ldhb*-M is similar to bovine *ldhb*, except for a few single nucleotide polymorphisms (Table 1). Although the amino acid substitutions are not located in substrate binding and catalytic sites of LDH (Figure 3), the shift of charge distribution in H subunits might still influence the properties of the three LDH1 phenotypes, such as *K*m values. The molecular modeling of yak LDH provided evidence to support the possible influence of LDH1 polymorphism on its catalytic property.

## 4. Experimental Section

### 4.1. Animals and Tissue Samples

One hundred and thirty adult Jiulong yaks were used for this study. The yaks were raised in Jiulong County of Sichuan Province, China, at 3500 meters of altitude, and grazed on natural pasture without feed supplementation. Within 30 min after slaughter, heart and *longissimus* muscles, at the position between the last thoracic spine and the third lumbar spine of the right carcass, were taken from each yak. All of the samples were promptly frozen and stored at −80 °C until analysis. In addition, a total of 32 whole milk samples of Zhongdian yak (a yak breed) were collected in Zhongdian, Yunnan province of China, and used for isolation of genomic DNA. Muscle samples were obtained in a slaughterhouse during approved commercial yak slaughter for meat, and milk samples were collected on a farm during normal milking process. All the experiments were conducted in China and followed the recommendations in the Guide for the Care and Use of Laboratory Animals of the National Institutes of Health. The animal use protocol was approved by the Experimentation Committee of Southwest University for Nationalities.

### 4.2. Muscle Extracts Preparation

Yak hearts and *longissimus* muscles (0.5 g each) were homogenized with electric homogenizer in 5 mL of 20 mmol/L Tris-HCl (pH 7.5), at 4 °C, and then centrifuged at 10,000× *g* for 20 min at 4 °C [18]. The supernatant was subjected to electrophoresis of LDH isozymes.

### 4.3. Electrophoresis of Yak LDH Isozymes

LDH isozymes from heart and *longissimus* muscles extracts were separated with native polyacrylamide electrophoresis (PAGE) using 7% separating gel. The electrophoresis was performed at 4 °C, and after electrophoresis, the gel was LDH activity stained according to the method reported [19]. The LDH1 phenotypes were identified by their different gel mobility. The relative percentage of each LDH isozyme was estimated by Quantity One software of Versa Doc 1000 gel imager system (Bio-Rad, Richmond, CA, USA).

### 4.4. Cloning and Sequencing of Yak *ldhb* Alleles

Total RNA was extracted from the heart tissues of Jiulong yaks carrying with different LDH1 phenotypes (LDH1-F, LDH1-M, and LDH1-S as identified by PAGE; three yaks for each phenotypes, *n* = 9) using TRIzol reagent (Invitrogen, Auckland, New Zealand) according to the manual instructions. The first strand cDNA was synthesized by TaKaRa RNA PCR Kit (AMV) Ver.3.0 kit using 1 μg total RNA in the presence of Oligo-dT primer following the manufacturer’s protocol. The PCR primers were designed based on the nucleotide sequence of bovine *ldhb* (GenBank accession number: AJ401268) to amplify the entire open reading frame of yak *ldhb* cDNA. The primers were as follows: *ldhb*-F: CAGCCCTTATCACTCTCTGGA, and *ldhb*-R: GCTAAGTGTCTACAGCCTTGCAG. The 1079bp amplified fragment was purified by gel purification kit and then cloned into pMD 18-T Vector (TaKaRa) for sequencing. At least five clones were sequenced for each yak carrying specific LDH1 phenotype, in order to detect possible single nucleotide polymorphisms in the *ldhb* sequence. The deduced amino acid sequences and protein properties of the *ldhb* were analyzed using online software [20].

### 4.5. Assay of *ldhb* G896A Mutation by PCR-RFLP in Two Yak Breeds

Since a mutation of G896A was detected in the Jiulong yak *ldhb* cDNA sequence (in exon 7), which contributes to the formation of LDH1-F phenotype, we detected its distribution in two yak breeds. Genomic DNA was isolated from *longissimus* muscles of Jiulong yaks using DNA isolation kit, or from the whole milk of Zhongdian yaks as described by other researchers [21] and stored at −20 °C. A pair of PCR primers was designed according to bovine *ldhb* DNA sequence (NC_007303.4): *ldhb*-e7-F: 5′-TGCAGTTCGTCCTGTTCAAC-3′; *ldhb*-e7-R: 5′-CAAGCATGGGCTTTGATTCT-3′. The expected PCR product was 402 bp, which was then digested with *Taq I* at 65 °C for 3 h and separated with 1.5% agarose gel electrophoresis. The G to A change at the 896 position of *ldhb* gene creates a *Taq I* digestion site, and therefore produces two fragments (256 bp and 146 bp).

### 4.6. Homology Modeling of H Subunit of Yak LDH

The three-dimensional structure of the H subunit of yak LDH was modeled by using Swiss-PdbViewer [22,23]. The templates used for modeling were human LDH subunit B (PDB accession number: 1i0zA).

## 5. Conclusions

Yak *ldhb* gene which encodes H subunit of LDH was obtained by RT-PCR. A total of six nucleotide differences were detected in yak *ldhb* compared with that of cattle, of which five mutations cause amino acid substitutions. The mutations of G896A and C689A in *ldhb* gene create the unique three phenotypes (F, M, and S) of yak LDH1. PCR-RFLP analysis of G896A mutation demonstrated that most LDH1-F samples are actually heterozygote at this site, which results in complicated bands of yak LDH isozymes during electrophoresis.

## Figures and Tables

**Table 1:** Nucleotide position differences in *ldhb* alleles of Jiulong yak.

Species	*ldhb*	53	204	407	689	700	896
Yak	F	G/A	T/C	G/T	A	A/G	A
	M	G/A	T/C	T	A	A/G	G
	S	G	C	T	C	A	G
Cattle		G	T	T	A	A	G

**Table 2:** Amino acid position differences in yak LDH1 phenotypes.

Species	LDH1 Phenotype	Amino acid position				

		17	135	229	233	298
Yak	F	Arg/Lys	Gly/Val	Glu	Val/Met	Gln
	M	Arg/Lys	Val	Glu	Val/Met	Arg
	S	Arg	Val	Ala	Met	Arg
Cattle		Arg	Val	Glu	Met	Arg

**Table 3:** Theoretical isoelectric point and molecular weight for H subunit of yak LDH1.

Species	Phenotype	pI	Molecular weight (Da)	GenBank accession No.
Yak	F	5.87	36564.39	HQ874652
	M	6.03	36592.45	HQ874653
	S	6.21	36534.41	HQ874654
Cattle	-	6.03	36592.45	BC102217

**Table 4:** Genotype and gene frequencies of *ldhb* G896A mutation in two yak breeds.

Breed	*n*	Genotype frequency			Gene frequency	
	
		AA	AG	GG	A	G
Jiulong yak	130	0.038 (5)	0.446 (58)	0.515 (67)	0.262	0.738
Zhongdian yak	32	0	0.375 (12)	0.625 (20)	0.188	0.812

The numbers in bracket are the sample number of corresponding genotypes.

## References

[1] Wiener G., Han J.L., Long R.J. (2003). The Yak.

[2] Markert C.L. (1984). Biochemistry and function of lactate dehydrogenase. Cell Biochem. Funct.

[3] Li S.S. (1989). Lactate dehydrogenase isoenzymes A (muscle), B (heart) and C (testis) of mammals and the genes coding for these enzymes. Biochem. Soc. Trans.

[4] Sheafor B.A. (2003). Metabolic enzyme activities across an altitudinal gradient: an examination of pikas (genus *Ochotona*). J. Exp. Biol.

[5] Amano T., Yamado W., Nabika T., Zhang X.L. (1990). Blood protein polymorphisms of Tibetan native cattle, yaks and their hybrid. Rep. Soc. Res. Nativ. Livest.

[6] Zheng Y.C., Zhao X.B., Zhou J., Piao Y., Jin S.Y., He Q.H., Hong J., Li N., Wu C.X. (2008). Identification of yak lactate dehydrogenase B gene variants by gene cloning. Sci. China Life Sci.

[7] Hoppeler H., Vogt M., Weibel E.R., Flück M. (2003). Response of skeletal muscle mitochondria to hypoxia. Exp. Physiol.

[8] Lin Y.Q., Wang G.S., Feng J., Huang J.Q., Xu Y.O., Jin S.Y., Li Y.P., Jiang Z.R., Zheng Y.C. (2011). Comparison of enzyme activities and gene expression profiling between yak and bovine skeletal muscles. Livest. Sci.

[9] Kuang L.D., Zheng Y.C., Lin Y.Q., Xu Y.O., Jin S.Y., Li Y.P., Dong F., Jiang Z.R. (2010). Studies on high altitude adaptation of yak based on genetic variants and activity of lactate dehydrogenase-1. Biochem. Genet.

[10] Zhang L., Ma B., Wu J., Fei C., Yang L., Wan H. (2010). Cloning and characterization of the yak gene coding for calpastatin and *in silico* analysis of its putative product. Acta Biochim. Pol.

[11] Bai W.L., Yin R.H., Zheng Y.C., Ma Z.J., Zhong J.C., Rin R.L., Dou Q.L., Zhang S.C., Luo G.B., Zhao Z.H. (2010). Cloning and molecular characterization of a yak α-lactalbumin cDNA from mammary tissue. Livest. Sci.

[12] Scheinfeldt L.B., Tishkoff S.A. (2010). Living the high life: High-altitude adaptation. Genome Biol.

[13] Avivi A., Gerlach F., Joel A., Reuss S., Burmester T., Nevo E., Hankeln T. (2010). Neuroglobin, cytoglobin, and myoglobin contribute to hypoxia adaptation of the subterranean mole rat *Spalax*. Proc. Natl. Acad. Sci. USA.

[14] Vogt M., Puntschart A., Geiser J., Zuleger C., Billeter R., Hoppeler H. (2001). Molecular adaptations in human skeletal muscle to endurance training under simulated hypoxic conditions. J. Appl. Physiol.

[15] Natarajan R., Fisher B.J., Fowler A.A. (2003). Regulation of hypoxia inducible factor-1 by nitric oxide in contrast to hypoxia in microvascular endothelium. FEBS Lett.

[16] Beall C.M. (2007). Two routes to functional adaptation: Tibetan and Andean high-altitude natives. Proc. Natl. Acad. Sci. USA.

[17] Gelfi C., de Palma S., Ripamonti M., Wait R., Eberini I., Bajracharya A., Marconi C., Schneider A., Hoppeler H., Cerretelli P. (2004). New aspects of altitude adaptation in Tibetans: A proteomic approach. FASEB J.

[18] Jurie C., Ortigues-Marty I., Picard B., Micol D., Hocquette J.F. (2006). The separate effects of the nature of diet and grazing mobility on metabolic potential of muscles from Charolais steers. Livest. Sci.

[19] Dietz A.A., Lubrano T. (1967). Separation and quantitation of lactic dehydrogenase isoenzymes by disc electrophoresis. Anal. Biochem.

[20] ExPASy SIB Bioinformatics Resource Portal http://www.expasy.org.

[21] Amills M., Francino O., Jansa M., Sanchez A. (1997). Isolation of genomic DNA from milk samples by using Chelex resin. J. Dairy Res.

[22] Arnold K., Bordoli L., Kopp J., Schwede T. (2006). The SWISS-MODEL Workspace: A web-based environment for protein structure homology modelling. Bioinformatics.

[23] Kiefer F., Arnold K., Künzli M., Bordoli L., Schwede T. (2009). The SWISS-MODEL repository and associated resources. Nucleic Acids Res.

